# Cellulose Synthase in Atacama Cyanobacteria and Bioethanol Production from Their Exopolysaccharides

**DOI:** 10.3390/microorganisms11112668

**Published:** 2023-10-30

**Authors:** Alexandra Galetović, Gabriel Peña, Nicole Fernández, Milton Urrutia, Nataly Flores, Benito Gómez-Silva, Jocelyne Di Ruggiero, Carolina Shene, Mariela Bustamante

**Affiliations:** 1 Laboratorio de Bioquímica, Departamento Biomédico, Facultad de Ciencias de la Salud, Universidad de Antofagasta, Av. Universidad de Antofagasta 02800, Campus Coloso, Antofagasta 1271155, Chile; gabriel.pena.pena@ua.cl (G.P.);; 2 Laboratorio de Genómica Microbiana, Departamento Biomédico, Facultad de Ciencias de la Salud, Universidad de Antofagasta, Av. Universidad de Antofagasta 02800, Campus Coloso, Antofagasta 1271155, Chile; 3 Centre for Biotechnology and Bioengineering, CeBiB, Beauchef 851, North Building—7th Floor, Santiago 8370456, Chile; 4 Millennium Institute Center for Genome Regulation, MI-CRG, Av. Libertador Bernardo O’Higgins No. 340, Santiago 8331150, Chile; 5 Ciencias Médicas, Facultad de Medicina y Odontología, Universidad de Antofagasta, Av. Argentina 2000, Antofagasta 1270001, Chile; 6 Department of Biology and Department of Earth and Planetary Sciences, Johns Hopkins University, Baltimore, MD 21218, USA; 7 Department of Chemical Engineering and Center of Food Biotechnology and Bioseparations, BIOREN, Universidad de La Frontera, Casilla 54-D, Temuco 4811230, Chile; 8 Scientific and Technological Bioresource Nucleus, BIOREN, Universidad de La Frontera, Casilla 54-D, Temuco 5468901, Chile

**Keywords:** cyanobacteria, cellulose, bioethanol, exopolysaccharides, cellulose synthase

## Abstract

Cyanobacteria produce exopolysaccharides (EPSs) as an adaptative mechanism against ultraviolet radiation and desiccation. Cellulose is present in the extracellular polymeric substance in some cyanobacteria genera and it has been proposed as a raw material for biofuel production. The goal of this work was to evaluate the cellulose presence in EPS of Atacama cyanobacteria strains and its use as an alternative and innovative biological source to produce bioethanol. The presence of cellulose was evaluated using techniques of molecular biology, bioinformatics, and electronic microscopy. The conserved motif D,D,D,35QXXRW, characteristic of processive β-glycosyltransferase in all cellulose-producing organisms, was identified in the genome of the LLA-10 strain. This is evidence that cellulose synthase in the LLA-10 strain is a functional enzyme. EPS from Atacama cyanobacteria was hydrolyzed by β-glucosidases (cellobiase and cellulase) and the released glucose was yeast-fermented to ethanol. Ethanol production reached 172.69 ± 0.02 mg ethanol/g EPS after 48 h of incubation. These results are the first step in the evaluation of EPS produced by native cyanobacteria isolated from northern Chile for future biotechnological applications such as the production of bioethanol.

## 1. Introduction

Cyanobacteria produce an extracellular polymer substance that can be attached to sheaths, capsules, and slimes or released into the environment. The extracellular substance contains proteins, glycoproteins, and a wide variety the exopolysaccharides (EPSs) composed of galactose, glucose, mannose, arabinose, ribose, fucose, glucuronic, and glucuronic acids, among others [1,2,3,4]. The synthesis of EPS plays an important role in adhesion to substrates, motility, and desiccation in extreme environments; in habitats with high UV radiation, the UV-A sunscreen pigment scytonemin is found strategically in the EPS at the cyanobacteria sheath [2,3,5,6,7].

The homopolysaccharide cellulose is found in EPS of cyanobacteria members of the genera *Gloeocapsa*, *Oscillatoria*, *Anabaena*, *Nostoc*, and *Scytonema* but does not have the structural complexity of biopolymers like hemicellulose or lignin, making the cyanobacterial cellulose easier to degrade [8,9].

The proposed mechanisms for assembling and exporting EPS in cyanobacteria are based on studies in bacteria and genes found in the cyanobacterial genomes. Genes for proteins involved in the Wzx/Wzy and Wzm/Wzt (ABC transporter) models have been reported in *Synechocystis* PCC 6803, which assemble and export EPS; also, the gene *Tvtll0007* from thermophilic *Thermosynechococcus vulcanus* encodes a functional cellulose synthase responsible for cellulose biosynthesis [10,11].

Cellulose synthase is the main enzyme that produces cellulose in plants, fungi, bacteria, archaea, and tunicates, among others, as a biopolymer composed of D-glucose monomers ligated by β (1–4) bonds [12,13,14,15,16,17]. In plants, cellulose biosynthesis is catalyzed by a cellulose synthase (CesA) complex assembled as hexameric rosettes at the plasma membrane that synthesizes chains of β-glucans that assemble as crystalline cellulose microfibrils [18,19]. The cellulose synthases of plants, bacteria, and cyanobacteria have a cytosolic catalytic domain with the D,D,D,35QXXRW motif needed for the enzyme activity. However, only cyanobacteria and plants have the specific Plant-Conserved Region (CR-P) within the catalytic domain of cellulose synthases [20].

Applications of cellulose can be found in medicine, cosmetics, and in biofuel generation as an alternative renewable energy source to fossil fuel. Ethanol production from sugar-rich biomass has been reported from plants, microalgae, and cyanobacteria [21,22]. Advantages of ethanol generation from cyanobacterial biomass over higher plants include high growth rates, minimal use of agricultural lands, lower pollution, chemically simpler polysaccharides, and lower production costs. Also, a large percentage of the dried cyanobacterial biomass contains glucose-rich polysaccharides, and its use in alcohol production has been extensively reported [23,24].

Currently, companies dedicated to the production of biofuel such as biodiesel or bioethanol and derivative products mainly use algae as raw material, some successful examples of which are Algenol, Alga Energy, and Biofuels Systems. Thus, our study contributes to the development of a biotechnological application of native cyanobacteria from the Atacama Desert where favorable natural conditions exist for its cultivation [25,26,27].

Additionally, learning on the adaptive strategies from microorganisms inhabiting hostile environments, such as the native cyanobacteria of the Atacama Desert, Chile, provides the opportunities to understand and apply this scientific knowledge in the medical, food, paper, and biofuel industries and, potentially, for sustainable sources of food, oxygen, and fuel necessary to explore and establish human colonies on Mars. Therefore, this work is focused on determining the presence of cellulose in the EPS of native cyanobacteria and its use as raw material for ethanol biosynthesis using biochemical, molecular, bioinformatics, and microscopic approaches for biotechnological applications.

## 2. Materials and Methods

### 2.1. Cyanobacteria Culture Conditions

Cyanobacterial strains LLA-10, LLC-10, PUT-08, RPU-10, QMA-10, LCHI-10, and CHU-08 were collected at pre-Andean and Andean wetlands from the Atacama Desert (located between 3900 and 4253 m.a.s.l.), Region of Antofagasta, Chile.

The purified strains were maintained at the Microbial Collection, Biochemistry Laboratory of the University of Antofagasta, in liquid (25–50 mL) and solid Arnon medium without combined nitrogen [28]. Cells were grown at 30 °C, under continuous white fluorescent light (180 μE m^−2^s^−1^), and airflow-enriched with 1% (*v*/*v*) CO_2_. Culture glass flasks (160–250 mL) were used to obtain a supernatant for EPS recovery as described below (Material and Methods, Section 2.4).

### 2.2. Extraction and Quantification of Genomic DNA

Genomic DNA was extracted using the UltraClean^®^ Microbial DNA Isolation Kit (Mo Bio Laboratories, Inc., Carlsbad, CA, USA). To facilitate cell disruption, 250 µL of TELT solution and 50 µL of lysozyme (3.25 mg/mL) were added and incubated at 37 °C for 45 min in a thermoregulated bath (Polyscience, Warrington, PA, USA) prior to DNA extraction. DNA quality was evaluated through gel electrophoresis (0.8% agarose) and visualized in a UV transilluminator (TS-40 UVP). DNA concentration was measured using absorbance at 260 nm, in spectrophotometer Shimadzu UV-1601 (Shimadzu Corporation, Tokyo, Japan). Finally, DNA was stored at −20 °C.

### 2.3. Molecular Study of Cellulose Synthase Gene

#### 2.3.1. PCR Amplification, Sequencing, and Similarity Searches for Cellulose Synthase Gene

Cellulose synthase gene was amplified using the primers CSSAF and CSSAR [10] and LLACELUF (5′CTCAGCAGATGCGAACA3′) and LLACELUR (5′AGGTGCTAATCCTTCGGC3′). The amplified PCR products were cloned into the pGEM-T easy vector (Promega Corporation, Madison, WI, USA) and transformed into *E. coli* strain DH-5α. The following reagents were used in the PCR reaction: 36 μL of sterile miliQ water, 1 μL of 10 mM dNTPs (dATP, dGTP, dCTP, and dTTP), 4 μL of 50 mM MgCl_2_, 5 μL of 10X PCR buffer, 1 μL of 120 pmoles/μL sense primer, 1 μL of 120 pmoles/μL antisense primer, 1 μL of 120 ng/μL DNA, and 1 μL of 5 U/μL Taq polymerase (Invitrogen, Carlsbad, CA, USA) in a final volume of 50 μL. For the amplification of the cyanobacterial cellulose synthase gene, the PCR reaction was performed in a thermocycler (TC-PRO, BOECO), using the following program: pre-denaturation at 95 °C for 4 min, followed by amplification for 35 cycles: 1 min at 95 °C, 1 min at 50 °C, 1 min at 72 °C, and final extension at 72 °C for 7 min. The amplified DNA was evaluated through 0.8% agarose gel electrophoresis. Sequencing and similarity searches were performed for nucleotide sequences of cellulose synthase. Plasmids containing the expected insert of 1,500 bp and 909 bp were sequenced at Macrogen, Korea, URL: http://ADN.macrogen.com/ (accesed on 14 July 2023). The cellulose synthase of *Nostoc* Llayta sp. sequence was deposited under accession number OR487487 in NCBI data bank. Searches were performed using BLAST [29]. Only alignments with an E-value between 0 and 1 × 10^−3^ were selected.

#### 2.3.2. Analysis of the Conserved Motif D,D,D,35QXXRW

Multiple alignments of cellulose synthase amino acid sequences were performed using the Muscle algorithm, version 5 URL: https://drive5.com/muscle5/manual/about_muscle5.html (accesed on 6 July 2023). The cellulose synthase sequences used in the alignments were the following: *Nostoc* sp. FACHB-973 (WP_190883645.1); *Nostoc* sp. ATCC 43529 (RCJ19960.); *Nostoc minutum* NIES-26 (RCJ36521.1); Nostocales cyanobacterium LEGE 11386 (MBE9051224.1); Unclassified *Nodularia* (WP_194001580); *Nodularia* sp. NIES-3585 (WP _089091285.1); *Microbacterium* sp. Ru50 (POX67145.1); *Rhodococcus equi* (ORM19494.1); *Sediminihabitans luteus* (GII98574.1); *Aspergillus niger* ATCC 13496 (RDH22901.1); *Penicillium brasilianum* (OOQ84387.1); *Penicillium chrysogenum* (KZN86950.1); *Pseudomonas* sp. GR 6-02 (ANI60103.1); *Treoboma cacao* (XP_017983276.1); *Stenotrophomonas panacihumi* (KRG45565.1); *Oscillatoria acuminata* (WP_015150828.1); *Chlorogloeopsis fritschii* (WP_016874772.1); *Synechocystis salina* (WP_194020110.1); *Xenococcus* sp. PCC 7305 (WP_006508287.1); *Ferroplasma acidiphilum* (ARD84887.1); and Archaeon (RLG68004.1) deposited in NCBI’s GenBank URL: http://www.ncbi.nlm.nih.gov/genbank (accesed on 6 July 2023). The deduced amino acid consensus sequence of 1500 bp for LLA-10 (483 aa) was obtained using ORF finder URL: http://www.ncbi.nlm.nih.gov/projects/gorf/orfig.cgi (accesed on 6 July 2023). The presence of the conserved motif of β-glycosyltransferases (D,D,D,35QXXRW) was analyzed using the GeneDoc program (version 2.7.000) URL: http://www.psc.edu/biomed/genedoc (accesed on 14 July 2023).

#### 2.3.3. Phylogenetic Analyses

A phylogenetic tree was constructed using the MEGA 11 program and the Neighbor-joining method, with a bootstrap of 5000 replicates [30], and the amino acid sequences of cellulose synthase catalytic subunits from the following organisms were deposited in NCBI’s GenBank URL: http://www.ncbi.nlm.nih.gov/genbank (accesed on 6 July 2023): *Nostoc* sp. FACHB-973 (WP_190883645.1); *Nostoc* sp. ATCC 43529 (RCJ19960.); *Nostoc minutum* NIES-26 (RCJ36521.1); *Nostocales cyanobacterium* LEGE 11386 (MBE9051224.1); *Unclassified Nodularia* (WP_194001580); *Nodularia* sp. NIES-3585 (WP _089091285.1); *Microbacterium* sp. Ru50 (POX67145.1); *Rhodococcus equi* (ORM19494.1); *Sediminihabitans luteus* (GII98574.1); *Aspergillus niger* ATCC 13496 (RDH22901.1); *Penicillium brasilianum* (OOQ84387.1); *Penicillium chrysogenum* (KZN86950.1); *Pseudomonas* sp. GR 6-02 (ANI60103.1); *Treoboma cacao* (XP_017983276.1): *Stenotrophomonas panacihumi* (KRG45565.1); *Oscillatoria acuminata* (WP_015150828.1); *Chlorogloeopsis fritschii* (WP_016874772.1); *Synechocystis salina* (WP_194020110.1); *Xenococcus* sp. PCC 7305 (WP_006508287.1); *Ferroplasma acidiphilum* (ARD84887.1); *Archaeon* (RLG68004.1); *Skeletonema marinoi* (KAK1745087.1); *Nitzschia inconspicua* (KAG7364622.1); *Fragilaria crotonensis* (KAI2505741.1); *Anopheles sinensis* (KFB35278.1); and *Lygus hesperus* (JAG29536.1). In addition, the deduced amino acid sequence of strain LLA-10 (483 aa) obtained with the NCBI, ORF Finder program URL: https://www.ncbi.nlm.nih.gov/orffinder/ (accesed on 6 July 2023) was included.

### 2.4. EPS Purification

Cyanobacterial cultures (250 mL) were centrifuged at 5000× *g* for 30 min, at 4 °C (RC SB Plus, 873 WIDB, Sorvall, Newtown, CT, USA), and EPS was recovered from the supernatants (160 to 200 mL) after precipitation with three volumes of absolute isopropyl alcohol through filtration under vacuum on sterile glass MGA glass-microfiber discs (Munktell, retention 1.6 µm; diameter 47 mm; pressure drop 38 mbar). After filtration, extensive washing was carried out with isopropyl alcohol/water (3:1 *v*/*v*) and dried at 70 °C for 48 h. The EPS was dissolved in distilled water to a final concentration of 5–10 mg/mL [31].

### 2.5. Enzymatic EPS Digestion and Fermentation

Twenty milligrams of the purified EPS from strain LLC-10 was digested at 37 °C for 1 h, with commercial cellulase from *Trichoderma reesei* (Celluclast) plus fungal cellobiase (Sigma, Burlington, MA, USA) in 0.1 M citrate buffer, pH 5.0, at a final assay volume of 10 mL.

Fermentation assays were conducted as follows: first, 10 mL of digested EPS was mixed with 5 mg of freeze-dried *Saccharomyces cerevisiae* URL: www.muntons.com (accesed on 28 May 2022); second, the reaction mix was incubated for four days at room temperature, under anaerobic conditions in glass flasks with pressure-resistant airlocks. Samples were retrieved at regular intervals (each 24 h) during 0–96 h to evaluate glucose or ethanol production.

### 2.6. Glucose and Ethanol Determination

Glucose produced after EPS fermentation was quantified using the glucose oxidase method described by Trinder (1969) [32], with the following modifications: aliquots (20 µL) from the EPS fermentation assay were mixed with 500 µL of a reaction mix containing 10.0 μL of glucose oxidase (1.5 KU/mL; diluted 1/100), 1.0 μL of peroxidase (1.0 KU/mL; diluted 1/1000), 33.0 µL of HBA (4-hidroxybenzoic acid; stock solution: 300 mM), 114.0 µL of 4 AAP (4-aminoantipyridine; stock solution: 22.8 mM), and 342.0 µL of 200 mM phosphate buffer, pH 7.5. The final solution was incubated for 10 min at 37 °C and the enzyme reaction was stopped by adding 50.0 µL of 0.4 M NaOH. Absorbance was measured at 500 nm in a UV-visible spectrophotometer (Shimadzu UV-1601, Shimadzu Corporation, Japan). A calibration curve was prepared with aliquots containing 0.0 to 20 µg of glucose (Sigma, Burlington, MA, USA) from a 0.25 µg/µL glucose standard solution.

Ethanol production was evaluated with alcohol dehydrogenase [33] with the following modifications: twenty microliters of the EPS-fermented samples were mixed with 14.0 μL of alcohol dehydrogenase (297 U/mL); 100 μL of 100 mM NAD+; 302 µL of a solution containing 248 mM sodium pyrophosphate, 248 mM semicarbazide, and 80 mM glycine, pH 8.8; and distilled water to a final volume of 1 mL. The assay was incubated for 30 min at 25 °C and the NADH generated was determined spectrophotometrically at 340 nm. A calibration curve was prepared with 0.0 to 3.75 μg of ethanol (Sigma, Burlington, MA, USA).

### 2.7. EPS Surface Morphology and Chemical Composition

Surface morphology of freeze-dried EPS (white powder) was characterized using the scanning electron microscope (SEM) SU3500 (Hitachi, Tokyo, Japan) at 12 kV with a 50 Pa vacuum and Backscattered Electron signal (BSE). The dry EPS was stored at 4 °C until microscopic analysis; the samples were dispersed over the sample holder equipped with a double-sided carbon tape. Energy-dispersive X-ray spectroscopy (EDX) using a QUANTAX 100 (Bruker, Mannheim, Germany) with a BSE detector, Mag 2000×, HV 10.0 KV, WD 10.8 mm, and Px 6.3 nm was used to determine the EPS chemical composition.

## 3. Results and Discussion

The LLA-10 strain was selected for the cellulose synthase study because we have previously studied and characterized it from a nutritional, toxicological, and ethnographic point of view [34,35,36]. Our interest was to extend this study to adaptive strategies such as the synthesis of EPS and other molecules with potential biotechnological applications of cyanobacteria isolated from unique environments in northern Chile.

### 3.1. Molecular Analysis

#### 3.1.1. Cellulose Synthase Gene Amplification

**Figure 1:**
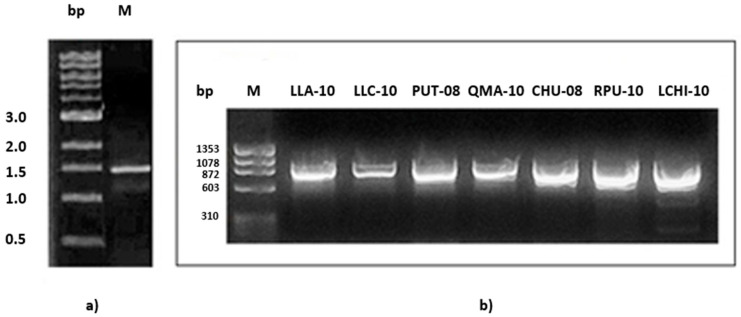
Amplification of the cellulose synthase catalytic subunit gene fragment. (**a**) The fragment obtained with the CcsAF and CcsAR primers had an expected size of 1500 bp, in the cyanobacterial strain LLA-10. A 1 kb molecular size marker was used. (**b**) Amplification of a fragment of the cellulose synthase catalytic subunit gene (909 bp) obtained with the LLACELUF-LLACELUR primers in all the cyanobacterial strains under study. The ϕX174 Hae III marker (1353–310 bp) was used.

Cellulose-producing organisms require cellulose synthase as an essential enzyme for cellulose biosynthesis from UDP-glucose to form β-1,4-glucan chains [37]. The presence of a putative gene coding for cellulose synthase in Atacama cyanobacteria was confirmed after amplification of two fragments (1500 and 909 bp) from the catalytic subunit of the cellulose synthase gene. The 1500 bp fragment was amplified from the genomic DNA purified from *Nostoc* sp. strain LLA-10, using primers CCSAF and CCSAR [9] (Figure 1a). The sequence of the 1500 bp fragment from strain LLA-10 was used to design new primers (LLACELUF and LLACELUR) that were used to screen the presence of the 909 bp fragment from the genomic DNA of Atacama cyanobacterial strains LLA-10, LLC-10, PUT-08, QMA-10, CHU-08, RPU-10, and LCHI-10 (Figure 1b). The use of these designed primers and sequencing of the PCR products allowed the identification of the 909 bp fragments from the genomic DNA of Atacama cyanobacterial strains LLA-10, LLC-10, PUT-08, QMA-10, CHU-08, RPU-10, and LCHI-10 (Figure 1b), confirming the presence of the cellulose synthase catalytic subunit gene in the isolated *Nostoc* strains from northern Chile. The sequenced PCR amplicons showed high similarity with the catalytic subunit of cellulose synthase of cyanobacteria: *Calothrix* sp. NIES-2100, *Nostoc carneum* NIES-2107, and *Anabaena* sp. WA102 (Table S1). The genera of cyanobacteria mentioned are representatives of the order Nostocales, which includes heterocyst-forming and filamentous cyanobacteria [38]. Alignments in the NCBI Database, using the 1500 bp amplicon as the query with blastn, blastx, tblastx, and the translated sequence of amino acids (483 aa) with blastp and tblastn, showed high identity (76–99%), coverage (96–100%), and reliability (E value 0) with cellulose synthase and glycosyl transferase in cyanobacteria of the genera *Nostoc*, *Anabaena*, *Calothrix*, and *Nodularia* (Table S1).

#### 3.1.2. Cellulose Synthase Catalytic Domains Alignment

**Figure 2:**
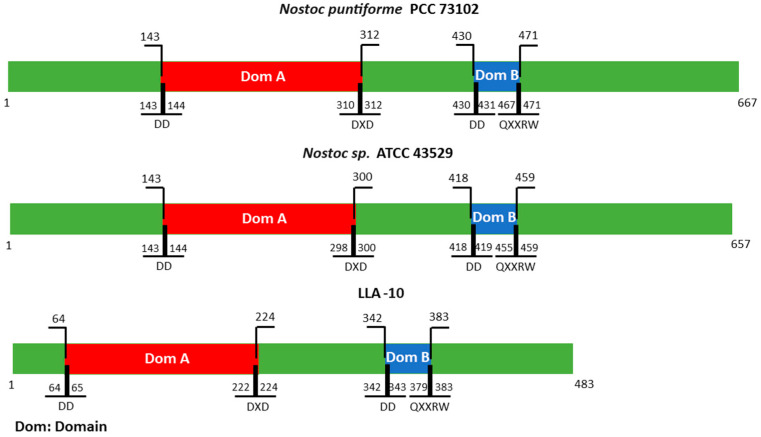
Schematic representation of the location of the conserved domains and D,D,D,35QXXRW motif in cyanobacteria. The cellulose synthase catalytic subunits of *Nostoc punctiforme* PCC 73102 (667 aa) and *Nostoc* sp. 43529 (657 aa) are compared to the 1500 bp fragment from strain LLA-10, translated to an amino acid sequence (483 aa). The locations of the conserved domain A (red) and B (blue) are indicated in the amino acid sequence (green) of each cyanobacteria.

The cellulose synthase catalytic subunit has two conserved domains and motifs. The positions of the conserved domains A and B in the genome Atacama strain LLA-10 with those of *Nostoc punctiforme* PCC73102 and *Nostoc* sp. ATCC43529 were compared (Figure 2). In the three cyanobacteria, the presence of the DD DXD motif was observed in domain A and the EDQXXRW motif in domain B (Figure 2). The presence of the D,D,D,35QXXRW motif is characteristic of active processive β-glycosyltransferase enzymes [39]. They are integral transmembrane proteins, widely distributed in prokaryotes, algae, amoebas, tunicates, and vascular plants; a synologic transfer of cellulose synthase genes from cyanobacteria to plants has even been proposed. The DXD, which is part of the conserved motif, is likely the UDP-glucose binding site and, therefore, important for enzyme functionality [9,37]. Variations in the amino acid sequence in this motif translated into non-functional enzymes, and site mutation directed to aspartic acid residues (D) in bacteria showed the loss of cellulose synthase enzymatic activity, emphasizing their catalytic importance [9,40,41]. Since the D,D,D,35QXXRW motif from strain LLA-10 did not show variations in its amino acid sequence, we concluded that LLA-10 cellulose synthase was a functional enzyme.

#### 3.1.3. Alignment of the Catalytic Region of Cellulose Synthase from Strain LLA-10

**Figure 3:**
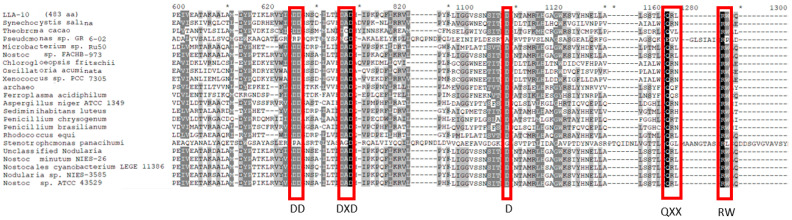
Alignment of the catalytic region of cellulose synthase LLA-10. The deduced amino acids sequence of LLA-10 was aligned with the catalytic region from diverse organisms. The conserved motif D,D,D,35QXXRW was identified (accession numbers are in Material and Methods Section 2.3). Amino acid residues are highlighted in black for 100%, dark gray for 80%, and light gray for 60% of identity.

The deduced amino acids sequence of the catalytic region of cellulose synthase from LLA-10 was aligned with those from diverse organisms in order to evaluate the presence or modifications of conserved motif D,D,D,35QXXRW. The DD position at domain A was conserved in strain LLA-10, as it is in plants *(Theobroma cacao*), fungi (*Penicillum* sp.), archaea *(Ferroplasma acidiphilum*), bacteria (*Microbacterium* sp.), and cyanobacteria (genera *Nostoc* and *Nodularia*). The DXD motif was present as DAD in strain LLA-10 and most of the organisms under analysis, except *Theobroma cacao* and the bacterium *Stenotrophomonas panacihumi* that have a DCD sequence (Figure 3). The QXX motif sequence showed higher variability among the organisms, but the sequence QRL in strain LLA-10 was coincident with that on cyanobacterial members of the genus *Nostoc* and *Nodularia.* In the case of the DXD motif, it was observed that *Theobroma cacao* presented a DCD and LLA-10 presented a DAD motif as in the majority of the organisms shown. There is a constant distance of 35 aa between residue D and the QXXRW motif in domain B of all cellulose-producing organisms (D35QXXRW) [20,42]. In the case of LLA-10, that distance was also 35 aa (Figure 2); however, a variation in the number of amino acid residues between the conserved amino acids of the motif D,D,D,35QXXRW would not affect the enzyme functionality as long as all the conserved residues were present. The data presented here therefore support the conclusion that strain LLA-10 is a cellulose producer.

#### 3.1.4. Phylogenetic Analysis with the Catalytic Region of Cellulose Synthase of Cyanobacterium Strain LLA-10

**Figure 4:**
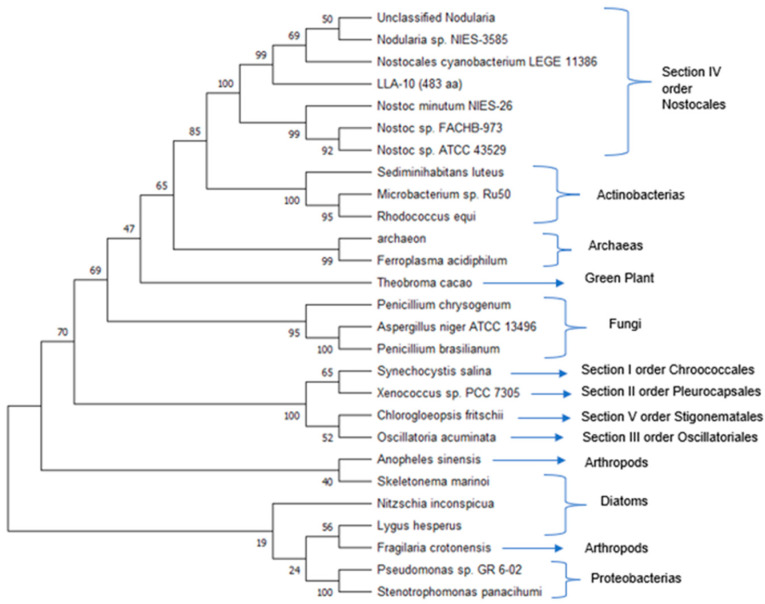
Phylogenetic tree of the catalytic subunit of cellulose synthase. The amino acid sequences of the 483 aa of catalytic subunit of cellulose synthase LLA-10, and other cyanobacteria, bacteria, fungi, diatoms, arthropods, and plants are shown. The phylogenetic tree was generated using the Neighbor-joining method and built considering a bootstrap of 5000 replicates. This phylogenetic analysis was performed using the Mega 11 program [30]. The accession numbers of the sequences included in the tree are described in Material and Methods (Section 2.3).

Deduced amino acid sequences of the cellulose synthase catalytic subunit from strain LLA-10 and other organisms were used to construct the phylogenetic tree shown in Figure 4. The amino acid sequence (483 aa) from strain LLA-10 was close to sequences from filamentous members of the genera *Nostoc* and *Nodularia* (Section IV, order Nostocales). Sequences from cyanobacteria belonging to sections I, II, II, and V from Actinobacteria, *Theobroma cacao* (green plant), fungi, proteobacteria, archaea, diatoms, and arthropods clustered independently. Phylogenetic analyses show that cellulose synthase amino acid sequences from various organisms are similar; cellulose biosynthesis is a common feature in cyanobacteria [8,19,43], cellulose synthase genes from eukaryotes may have derived from cyanobacteria by a multilateral gene transfer [8,44,45,46], and cellulose biosynthesis is similar in prokaryotes and eukaryotes and is evidence of an evolutionary link among cellulose-producing organisms [9,45,47].

### 3.2. Enzymatic EPS Degradation and Fermentation

#### 3.2.1. EPS Content among Cyanobacteria Strains

**Figure 5:**
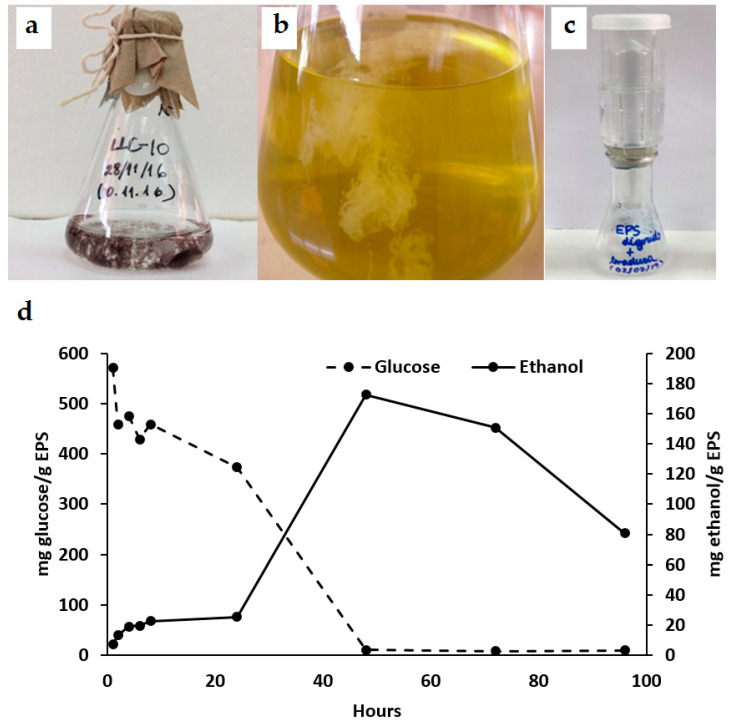
Kinetics of glucose fermentation and ethanol production. Strain LLC-10 was grown in liquid Arnon medium [28], at 30 °C, under white fluorescent light (180 μE m^−2^ s^−1^), with a continuous airflow enriched with 1% (*v*/*v*) CO_2_ (**a**). EPS was precipitated from the medium supernatant by isopropanol (**b**). EPS was fermented in an air-lock fermentation system, anaerobically with 0.5 mg/mL of a yeast suspension during four days at room temperature (**c**). Fermentations were conducted in triplicate in two independent experiments and glucose and ethanol production were measured at regular intervals (**d**).

Cyanobacteria-producing EPSs form a mucilaginous layer that contains cellulose without the structural complexity of hemicellulose or lignin; this cellulose can be degraded more easily than polysaccharides from plants [1,8]. Cyanobacteria, with a high carbohydrate content, are engaging candidates for bioethanol production because of lower production costs and decreased environmental pollution [22]. Cyanobacterial strains isolated from the Andes wetlands showed substantial differences in their EPS content (Table 1). All were filamentous diazotrophic cyanobacteria assigned to the *Nostoc* genus. Strain LLC-10 had the highest EPS content (7.1 g EPS per liter) and was selected for further analyses. LLC-10 EPS was extracted through isopropanol extraction, dried, and solubilized at a proper concentration for enzymatic EPS degradation and fermentation experiments (Table 1; Figure 5a–c).

#### 3.2.2. Fermentation and Ethanol Production from Cyanobacteria EPS

The chemical composition of microalgae and cyanobacteria includes 30–50% proteins, 8–15% lipids, and 20–50% carbohydrates [24,34]. Since 90% of the glucose content in *Synechococcus* sp. PCC 7002 biomass can be converted to ethanol [48], cyanobacterial biomass with high sugar content presents alternative raw materials for fermentation and ethanol production. EPS from strain LLC-10 was subjected to enzymatic digestion with commercial fungal β-glucosidases to increase glucose release for bioethanol production.

After the enzymatic hydrolysis of EPS (2 mg/mL), a seven-fold increase in ethanol production was observed during the 24–48 h of incubation, reaching a maximum of 172.69 ± 0.02 mg ethanol/g EPS, with a complete fermentation of the available glucose (Figure 5d). A decline in ethanol production occurred after 48 h (Figure 5d); ethanol volatilization or yeast alcohol dehydrogenase activity involvements may be considered as preliminary explanations for such an observation; it is an important fact that becomes a central point for selecting the right timing for ethanol extraction that must be studied for future applications.

It has been reported that the ethanol yield from cyanobacterial polysaccharides can be improved through various pre-treatments such as acid and enzymatic hydrolysis and supercritical fluid extraction [49,50]. Comparatively, total biomass fermentation in *Synechococcus* sp. PCC 7002, with 60% of its cell dry weight as carbohydrate, rendered 270 mg of ethanol per gram of cell dry weight [48], while ethanol production from fermented dried cells of green algae and cyanobacteria reported 210–350 mg of ethanol per gram of dry cell weight [51]; previously, fermentation of *Spirulina* glycogen yielded 500 mg of ethanol per gram [52]. These examples compare reasonably with the ethanol production obtained in this study with hydrolyzed EPS from Atacama strain LLC-10.

Future work on the EPS enzymatic digestions should consider (a) using other polysaccharide hydrolases to improve glucose release, and (b) modifications on the fermentation assays, e.g., pH, yeast concentration, and salt composition. Reports on ethanol production used whole cells as a substrate for yeast fermentation; however, the ethanol yield from cyanobacterial polysaccharides can be improved through pretreatments with acid, enzymatic hydrolysis, and supercritical fluid extraction [24,49,50].

To the best of our knowledge, our work is the first evidence of ethanol production from EPS obtained from an Atacama cyanobacterium. The strain LLC-10 is a raw material containing EPS with cellulose-like molecules and other sugars that can be degraded to glucose by β-glucosidases and be fermented to ethanol. These results are the first step in evaluating native cyanobacteria collected at the Atacama Desert as a biotechnological resource for future applications, such as biofuel production.

#### 3.2.3. Microscopy Analysis of Cyanobacteria EPS

**Figure 6:**
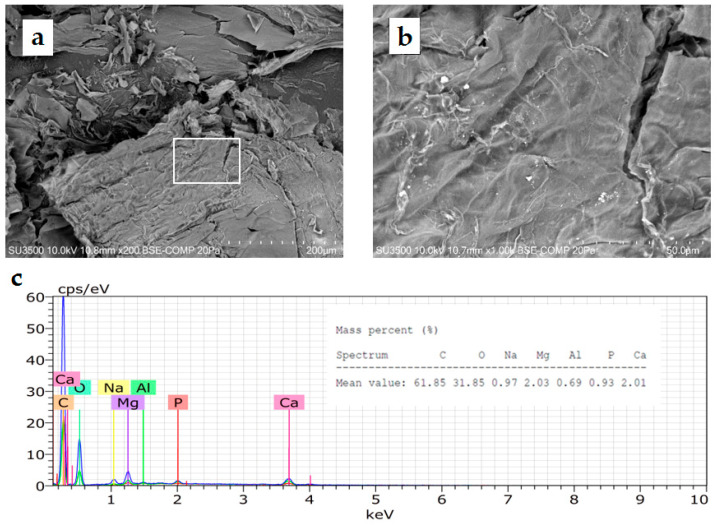
SEM micrographs (**a**,**b**) and energy-dispersive X-ray (EDX) analysis spectrum (**c**) of freeze-dried EPS isolated from cyanobacterial strain LLC-10.

The freeze-dried EPS of strain LLC-10 exhibited a compact structure with the appearance of a smooth surface without pores and filament-like structures (Figure 6a,b) resembling the microfibrils shown by TEM in *N. muscorum* UTEX 2209 [8]. Chemical composition analysis (Figure 6c) via EDX revealed the presence of C (61.85%), O (31.85%), Na (0.97%), Mg (2.03%), Al (0.69%), P (0.93%), and Ca (2.01%) expressed as mass percent (%). The high carbon and oxygen contents were partial evidence of the presence of polysaccharides in the Atacama EPS because the sugar fraction commonly found in cyanobacterial EPS includes glucose, galactose, mannose, fructose, ribose, xylose, and others [53].

## 4. Conclusions

EPSs purified from cyanobacterial strains isolated from wetlands at the Atacama Desert highlands in northern Chile, include cellulose-like molecules. Among different isolates, the Atacama cyanobacteria LLC-10 was the strain with the highest EPS content. A putative cellulose synthase gene was identified in the genome of the LLA-10 strain, and its amino acid sequence contained the conserved motif D,D,D,35QXXRW, characteristic in β-glycosyltransferases from all cellulose-producing organisms. Ethanol was generated after fermentation of β-glucosidase-hydrolyzed EPS from strain LLC-10, demonstrating the Atacama microbial richness and its potential for renewable bioenergy resources. Our results suggest that EPS from native cyanobacteria can be used as an alternative and innovative biological source to produce bioethanol and other biotechnological applications.

## Figures and Tables

**Table 1:** Extracellular polymeric substance content of cyanobacterial strains isolated from wetlands at the Andes Range in northern Chile.

Strain	EPS (g L^−1^)
LLA-10	2.65 ± 0.13
LLC-10	7.11 ± 0.02
RPU-10	1.84 ± 0.05
LCHI-10	1.73 ± 0.23

## Data Availability

The cellulose synthase of *Nostoc Llayta* sp. sequence was deposited under accession number OR487487 in NCBI data bank.

## Supplementary Materials

The following supporting information can be downloaded at https://www.mdpi.com/article/10.3390/microorganisms11112668/s1, Table S1: Alignment of the consensus sequence from cyanobacterial strain LLA-10.
