# Supplementary material for: Cellulose Synthase in Atacama Cyanobacteria and Bioethanol Production from Their Exopolysaccharides
Source: Microorganisms. 2023 Oct 30;11(11):2668. doi: 10.3390/microorganisms11112668 (PMC10673042; doi:10.3390/microorganisms11112668)
Supplement: Supplementary file 1 [file microorganisms-11-02668-s001.zip › microorganisms-2622993-supplementary.pdf]

Table S1. Alignment of the consensus sequence from cyanobacterial strain LLA-10. The nucleotides consensus sequence (1500 bp) and translated consensus aminoacid sequence (483 aa) were used as query in the blastn, blastx, tblastx, tblastp and tblastn aligments programs of NCBI. The table shows identity percentage, organisms and names for gene or proteins found with identity between 76–99%.

	Query	Alignment	E-value	Query cover	Percent Identity	Organisms	N° of access in GenBank	Description of the gene or protein
blastn	1500 pb	Nucleotide	0.0	99%	76,13%–77,05%	Calothrix sp. NIES-2100	AP018178.1	Cellulose synthase
						Nostoc carneum NIES-2107	AP018180.1	Cellulose synthase
						Anabaena sp. WA102	CP011456.1	Cellulose synthase
						Dolichospermum sp. DET69	CP070233.1	Glycosyl transferase
						Aphanizomenon flos-aquae DEX18	CP051188.1	Glycosyl transferase
						Nostoc linckia NIES-25	AP018223.1	Cellulose synthase
blastx	1500 pb	Nucleotide	0.0	99%	81,04%–99,79%	Nostoc minutum NIES-26	RCJ36521.1	Cellulose synthase
						Unclassified Nodularia	WP_194001580	Glycosyl transferase
						Nostocales cyanobacterium LEGE 11386	MBE9051224.1	Glycosyl transferase
						Nodularia sp. NIES-3585	WP_089091285.1	Glycosyl transferase
						Nostoc sp. ATCC 43529	RCJ19960.1	Cellulose synthase
						Nostoc sp. FACHB-973	WP_190883645.1	Glycosyl transferase
tblastx	1500 pb	Nucleotide	0.0	96%–99%	84,94%%–86,32%	Nostoc sp. NIES-2111	AP018184.1	Cellulose synthase catalytic subunit
						Calothrix sp. NIES-2100	AP018178.1	Cellulose synthase
						Calothrix sp. NIES-2098	AP018172.1	Cellulose synthase catalytic subunit
						Nostoc sp. NIES-3756	AP017295.1	Cellulose synthase catalytic subunit
						Nostoc linckia NIES-25	AP018223.1	Cellulose synthase
						Nostoc sp. C052	CP040272.1	Glycosyl transferase
blastp	483aa	Protein	0.0	96%–99%	81,04%–99,79%	Cyanocohniella sp. LLY	MCG6133374	Glycosyl transferase
						Unclassified Nodularia	WP_194001580	Glycosyl transferase
						Nostocales cyanobacterium LEGE 11386	MBE9051224.1	Glycosyl transferase
						Nodularia sp. NIES-3585	WP_089091285.1	Glycosyl transferase
						Nostoc sp. FACHB-973	WP_190883645.1	Glycosyl transferase
						Nostoc sp. ATCC 43529	RCJ19960.1	Cellulose synthase
tblastn	483aa	Protein	0.0	99%–100%	78,44%%–80,21%	Nostoc sp. NIES-3756	AP017295.1	Cellulose synthase catalytic subunit
						Nostoc sp. NIES-2111	AP018184.1	Cellulose synthase catalytic subunit
						Calothrix sp. NIES-2098	AP018172.1	Cellulose synthase catalytic subunit
						Nostoc linckia NIES-25	AP018223.1	Cellulose synthase
						Calothrix sp. NIES-2100	AP018178.1	Cellulose synthase
						Nostoc sp. C052	CP040272.1	Glycosyl transferase
